# Compact component for integrated quantum optic processing

**DOI:** 10.1038/srep16276

**Published:** 2015-11-20

**Authors:** Partha Pratim Sahu

**Affiliations:** 1 Department of Electronics and communication engg, Tezpur University, Assam, India

## Abstract

Quantum interference is indispensable to derive integrated quantum optic technologies (1–2). For further progress in large scale integration of quantum optic circuit, we have introduced first time two mode interference (TMI) coupler as an ultra compact component. The quantum interference varying with coupling length corresponding to the coupling ratio is studied and the larger HOM dip with peak visibility ~0.963 ± 0.009 is found at half coupling length of TMI coupler. Our results also demonstrate complex quantum interference with high fabrication tolerance and quantum visibility in TMI coupler.

Photonic technology has become a natural choice *for* optical quantum computing^1^, quantum optic processing^2^,^3^ optical quantum sensing^4^ and quantum communication security based on quantum entanglement^5^, because of *inherent* low noise, high speed and fidelity quantum interferences. In this direction, the use of bulk quantum optics has many practical problems such as reliability, robustness, size and precision^5^. It is also difficult to achieve optical phase control accuracy required for quantum information processing by using bulk quantum optics. To overcome the limitations, integrated optical waveguide concept has been introduced by Politi *et al.*^6^ with a number of advantages such as improved reliability, immunity to vibration and electromagnetic interference, low loss transmission, small size, light weight, low power consumption, and batch fabrication economy^5^,^7^,^8^.

Quantum optic logic gates are key devices for integrated optical quantum processors^9^,^10^ in which directional coupler (DC)^11^,^12^ and multimode interference (MMI) coupler^13^,^14^ are used as a basic component of integrated quantum optic circuits in which the essential requirement to derive quantum technology is quantum interference. Recently, it is reported^13^ that MMI devices show complex high fidelity quantum interference behavior through self imaging principle in which input field is reproduced in single or multiple images at periodic intervals along the propagation direction of MMI waveguide and Hong Ou Mandel (HOM) dip is also observed in 2 × 2 MMI coupler by same researchers. Further, MMI devices show excellent tolerances to polarizations and wavelength variations^14^. But, the present approach is to find compact basic waveguide device components for large scale integrated quantum optic processors. In this direction, we have already shown^8^,^15^ that due to having fewer number of waveguide parameter, two mode interference coupler has higher fabrication tolerance than MMI coupler. Further two mode interference coupler is more compact than MMI coupler because of lower coupling efficiency of higher order modes excited in MMI region than lower order modes (fundamental mode and first order mode which are only excited in TMI region)^16^. As the coupling length decreases slowly with increase of index contrast (∆n) for ∆n >0.05^8^,^15^, with small variation of n_1_ and n_2_ due to fabrication error, the change of coupling of modes (over/under coupling) is not affected much.

Two mode interference (TMI) coupler shown schematically in Fig. 1a is treated as a 2 × 2 beam splitter represented by the following transition matrix providing input to output field transition with quantum mechanical amplitudes for connections of input and output states.


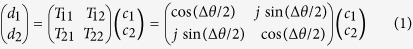


where T_11_, T_12_, T_21_ and T_22_ are matrix elements depending on phase changes of two excited modes (fundamental mode and first order mode) propagated through TMI region of length L. The phase difference 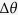 is written as 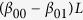, where, 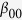 and 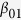 are propagation constants of fundamental mode and first order mode respectively. The special characteristic of the linear quantum device is that the coupling between two photons is achieved by using quantum interference. In this direction, the state of two photons in which one is in input c_1_ and other photon is in input c_2_ is described as 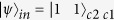. Ideally the maximally path entangled state of two photons at the output of TMI coupler is written as, 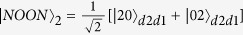 with probability of having this state in the output of TMI coupler 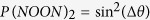. If the photons are entered through two input ports c_1_ and c_2_, the coincidence probability for detecting one photon in each of the outputs d_1_ and d_2_ is written as^16^,^17^, 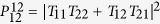. Quantum interference arises due to the indistinguishability of the photons depending mainly on polarization and arrival time. As TMI coupler is polarization independent, the polarization states of two photons in TMI region are *considered* to be same. Here, the indistinguishability of two photons is studied by varying arrival time of the photons which is taken to be parametric down conversion anti-correlated photons following *Gaussian* spectral density distribution^18^,^19^. Since, the coincidence measurement corresponds to coincidence probability 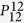, the expected number of the photon coincidence is then written as


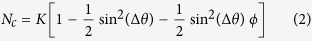


where, 
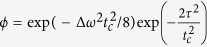
 = overlap integral representing degree of distinguishability of two photons^18^, 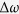 = difference between two central frequencies of *Gaussian* spectral distribution, 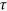 = Δx/c = time lag between the arrivals of two photons at the ports of TMI coupler and *t*_c_ = coherence time which corresponds to coherence length *l*_*c*_* = c.t*_*c*_ and K is a constant which is determined from incident number photons. For quantum mechanically indistinguishable photons (*τ* → 0), the expected number of the photon coincidence is written as,


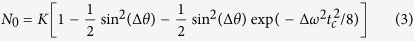


For classically distinguishable (τ → 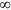), the photon coincidence, 
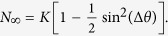


Depending on phase 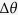, the photon coincidence is obtained for different beam splitting ratio of TMI coupler. After obtaining coincidence counts theoretically for quantum mechanically indistinguishable photons and classically distinguishable photons, we have demonstrated quantum interference in compact two mode interference coupler fabricated by using SiON/SiO_2_ technology. Here, we have also estimated HOM dip visibilities varying with coupling length of TMI coupler.

## Results

**Figure 1:**
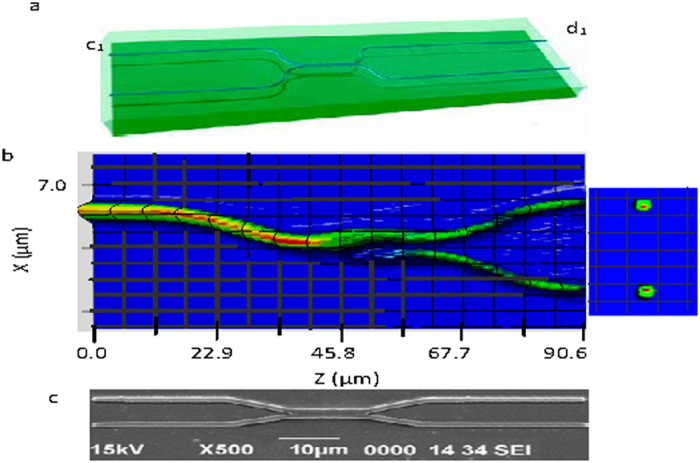
TMI devices. (**a**) Schematic diagram of 2 × 2 TMI integrated chip (**b**) classical light propagation is shown in the device where (**a**). light of wavelength 804 nm is launched in the input waveguide c_1_ and two mode propagation in TMI region results in equal intensity in each of the output, Analogous behaviour is observed for launching of light in waveguide c_2_. Output of the device confirms single mode light (**c**) SEM photograph of 2 × 2 3dB TMI coupler.

The 2 × 2 TMI couplers of different beam splitting ratio were designed with index contrast ∆n = 0.05 and core index of 1.5 by using a commercial available BPM package (Fig. 1b shows designed 3dB 2 × 2 TMI coupler). For all the designs and simulations, a wavelength of 0.804 μm and TE mode operation are taken. The deviation of design based on TM mode from that based on TE mode is within 0.25%, because of polarization independent property of TMI device. These devices were fabricated by using SiO_2_/SiON material. Figure 1c shows SEM photograph of 50:50 TMI coupler with coupling length ~11.5 μm.

### Two-photon quantum interference

**Figure 2:**
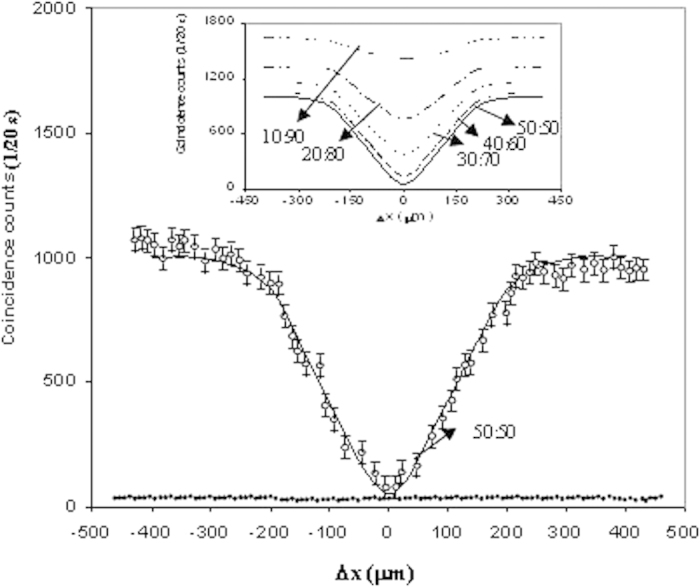
The measured HOM dip of 3 nm filter providing FWHM of 190 μm for 50:50 TMI coupler when inputting 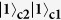. The solid line obtained theoretically by using the equation (3) for 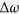 = 0.986 × 10^12^ s^−1^, λ = 0.804 μm, n_1_ = 1.5, n_2_ = 1.45 and w = 1.5 μm also represents same evidence. The figure also shows coincidence versus ∆x obtained theoretically by using the equation (3) for TMI coupler with splitting ratio 40:60, 30:70, 20:80 and 10:90. The degree of quantum interference for 40:60 splitting ratio is almost close to that of 50:50 splitting ratio.

Figure 2 shows the coincidence count rate versus relative delay in arrival time of photons (represented in terms of ∆x), measured simultaneously at both outputs d_1_ and d_2_ of TMI coupler by using the detectors with computer control. The Hong Ou Mandel (HOM) dip^20^ is found and centered on zero path difference, confirming occurrence of quantum interference. The behaviour of HOM dip varying with splitting ratio is well explained theoretically *in the figure*. The *largest and lowest HOM dip* are obtained (theoretically with the equation 3) for TMI coupler with splitting ratio 50:50 and 10:90 respectively. The degree of quantum interference is quantified by quantum interference visibility V which is written as


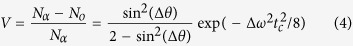


**Figure 3:**
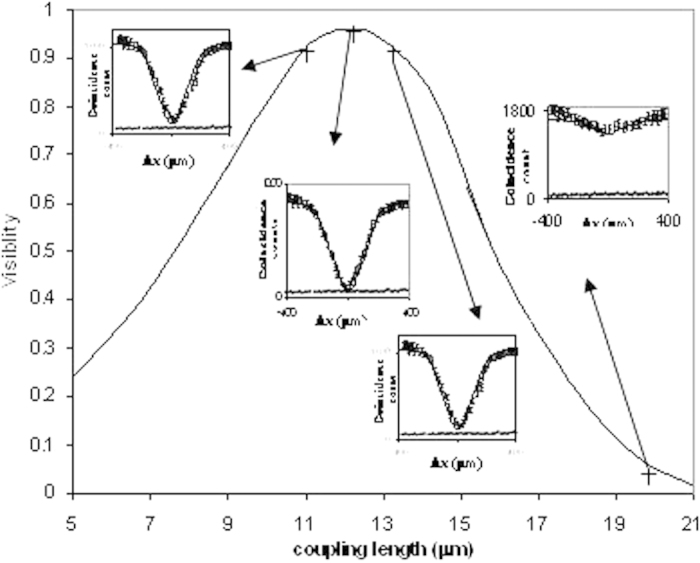
Quantum interference visibility of HOM dip experiments (cross sign s are experiment points) performed by using 2 × 2 TMI coupler with different coupling lengths (corresponding coupling ratios ~80:20, 60:40, 50:50, 40:60, 30:70 and 10:90) when inputting the photon state 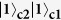. The solid line obtained theoretically by using the equation (4) for 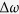 = 0.986 × 10^12^ s^−1^, λ = 0.804 μm, n_1_ = 1.5, n_2_ = 1.45 and w = 1.5 μm is almost close to the experimental results. The insets of the figure also shows coincidence versus ∆x obtained experimentally for TMI coupler with splitting ratio ~50:50 and 10:90. The dotted lines inside the insets shows the measured rate of accidental counts at dip minimum position.

Figure 3 shows Quantum interference visibility versus coupling length (corresponded to coupling ratio varying from 80:20 to 10:90) for 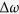 = 0.986 × 10^12^ s^−1^, λ = 0.804 μm, n_1_ = 1.5, n_2_ = 1.45 and w = 1.5 μm obtained by using the equation (4). It is seen that the peak visibility V obtained at half power length of 11.5 μm is 0.963 ± 0.009 which is more than that of 2 × 2 MMI device demonstrated by previous authors^13^. This is due to the fact that the jitter produced in TMI coupler is less than that of MMI coupler as *fewer numbers* of modes (2 modes) is propagated in TMI coupler than that of MMI coupler. *These results shows* high visibility quantum interference occurred in the TMI coupling length of 11.5 μm which is 90 times lower than that of MMI coupler. The figure also confirms high visibility quantum interference even with small variation of coupling ratio near to peak region of visibility.

**Figure 4:**
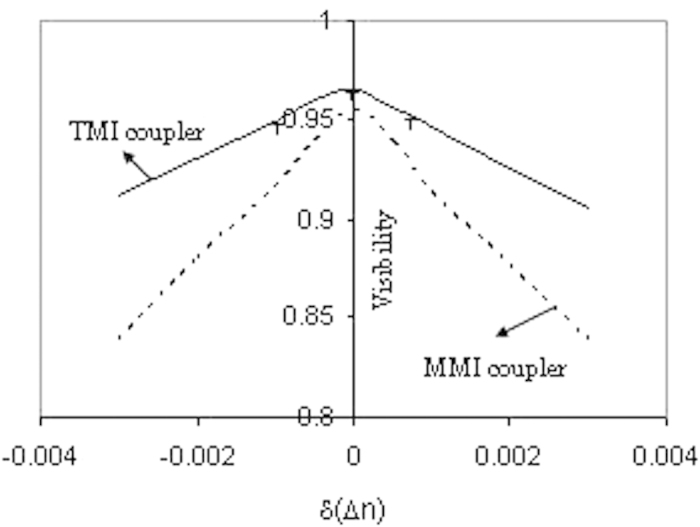
Quantum interference visibility versus small deviation (±δ(∆n)) of index contrast a due to fabrication errors obtained theoretically by using the equation (4) for 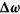 = 0.986 × 10^12^ s^−1^, λ = 0.804 μm, n_2_ = 1.45 and w = 1.5 μm. The solid line represent for 2 × 2 TMI coupler of half coupling length = 11.5 μm where as the dashed line indicates for 2 × 2 MMI coupler of half coupling length = 1035 μm. There is a slight degradation of quantum interference visibility with small variation of ∆n due to fabrication errors for TMI coupler in comparison to MMI coupler. The cross sign in the figure shows experimental values of reduction of visibility matching well with theoretical values.

For bending loss <0.01 dB^21^, the transition length L_T_ of the access waveguide (along z direction) having bending radius R and separation 2 H_T_ between access waveguides of TMI coupler (Fig. 1) is obtained as 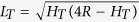
~ 44.4 μm (where R = 100 μm and H_T_ = 5 μm). Experimental measurement confirmed the insertion loss ~1.5 dB obtained by considering same transition length of the access waveguide. *The overall length* of 50:50 TMI beam splitter is obtained as ~100.8 μm which is ~29.5 times less than that of directional coupler reported by previous authors^22^. Due to fabrication errors, there may be slight deviation of core and cladding refractive index which leads to degradation of quantum interference visibility in 50:50 TMI coupler and 50:50 MMI coupler. Figure 4 shows less quantum interference visibility reduction in TMI coupler than that in MMI coupler.

TMI coupler has not only offered as an ultra compact component but also a high quantum interference visibility component for large scale quantum optic circuit. The TMI couplers promise to develop miniaturization and prototyping of complex quantum logic devices. Due to having *fewer number* of fabrication parameters, TMI couplers of splitting ratio from 50:50 to 40:60 provides almost same high quantum interference visibility. So this paves practical way for miniaturizing, scaling, and improving the performance for future quantum optic processing and network.

## Methods

### Devices

On silicon substrate, embedded waveguide having TMI *region* of width 2w ~ 3 μm and length ~12 μm with silicon oxinitride core of refractive index 1.5 and silica cladding of refractive index 1.45 were fabricated on silicon substrate by combination of plasma enhanced chemical vapor deposition (PECVD), photolithography and reactive ion etching (RIE) and. The overall length of the chip from input to output was ~100.8 μm.

### Experiment

The quantum interference experiments were performed by launching two photons into inputs (one into c_1_ and other into c_2_), generated by spontaneous down parametric conversion crystal made of type-I phase matched bismuth borate (BiB_3_O_6_) pumped with 0.402 μm wavelength pulse laser diode. The photon pairs made by BiB_3_O_6_ crystal are traveled through 3 nm interference filter which allows each photon with coherence length of *l*_*c*_* = λ*^2^*/*Δ*λ ~ *215 μm. The photon were collected from the device outputs into butt-coupled single mode polarization maintaining fiber (PMF) and coupled to silicon single photon avalanche photodiodes (APDs)^13^. In case of low average pump power the state 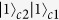
was produced at rate of 100 s^−1^ for two photon quantum interference.

## Additional Information

**How to cite this article**: Sahu, P. P. Compact component for integrated quantum optic processing. *Sci. Rep.* **5**, 16276; doi: 10.1038/srep16276 (2015).
